# The Impact of Meat Intake on Bladder Cancer Incidence: Is It Really a Relevant Risk?

**DOI:** 10.3390/cancers14194775

**Published:** 2022-09-29

**Authors:** Achille Aveta, Crescenzo Cacciapuoti, Biagio Barone, Erika Di Zazzo, Francesco Del Giudice, Martina Maggi, Matteo Ferro, Daniela Terracciano, Gian Maria Busetto, Giuseppe Lucarelli, Octavian Sabin Tataru, Emanuele Montanari, Benito Fabio Mirto, Alfonso Falcone, Gaetano Giampaglia, Enrico Sicignano, Federico Capone, Gianluca Villano, Pasquale Angellotto, Celeste Manfredi, Luigi Napolitano, Ciro Imbimbo, Savio Domenico Pandolfo, Felice Crocetto

**Affiliations:** 1 Department of Neurosciences, Reproductive Sciences and Odontostomatology, University of Naples Federico II, 80131 Naples, Italy; 2 Department of Precision Medicine, University of Campania “Luigi Vanvitelli”, 80138 Naples, Italy; 3 Department of Medicine and Health Sciences “V. Tiberio”, University of Molise, 86100 Campobasso, Italy; 4 Department of Maternal Infant and Urologic Sciences, Policlinico Umberto I Hospital, “Sapienza” University of Rome, 00161 Rome, Italy; 5 Division of Urology, European Institute of Oncology IRCCS, 20141 Milan, Italy; 6 Department of Translational Medical Sciences, University of Naples “Federico II”, 80131 Naples, Italy; 7 Department of Urology and Organ Transplantation, University of Foggia, 71122 Foggia, Italy; 8 Urology, Andrology and Kidney Transplantation Unit, Department of Emergency and Organ Transplantation, University of Bari, 70124 Bari, Italy; 9 The Institution Organizing University Doctoral Studies (I.O.S.U.D.), George Emil Palade University of Medicine, Pharmacy, Sciences and Technology, 540142 Târgu Mureș, Romania; 10 Department of Urology, Fondazione IRCCS Ca’ Granda Ospedale Maggiore Policlinico, Via della Commenda 15, 20122 Milan, Italy; 11 Department of Clinical Sciences and Community Health, University of Milan, 20122 Milan, Italy; 12 Urology Unit, Department of Woman Child and of General and Specialist Surgery, University of Campania “Luigi Vanvitelli”, 80121 Naples, Italy; 13 Division of Urology, VCU Health, Richmond, VA 23298, USA

**Keywords:** bladder cancer, red meat, processed meat, white meat, prevention, diet, carcinogenesis

## Abstract

**Simple Summary:**

Public health is severely challenged by bladder cancer (BC). There are few known risk factors for BC, but among these, diet seems to have a potential role in its etiology. However, the effect of meat is yet to be determined. In this study, we reviewed the influence of meat consumption on BC incidence. Our results showed different associations between meat consumption and BC depending on the type of meat consumed, the cooking method and the temperature used. Consequently, the promotion of healthy lifestyle interventions based on dietary factors can halt BC onset and can ameliorate the treatment efficacy of BC patients.

**Abstract:**

Bladder cancer (BC) represents the second most common genitourinary malignancy. The major risk factors for BC include age, gender, smoking, occupational exposure, and infections. The BC etiology and pathogenesis have not been fully defined yet. Since catabolites are excreted through the urinary tract, the diet may play a pivotal role in bladder carcinogenesis. Meat, conventionally classified as “red”, “white” or “processed”, represents a significant risk factor for chronic diseases like cardiovascular disease, obesity, type 2 diabetes, and cancer. In particular, red and processed meat consumption seems to increase the risk of BC onset. The most accepted mechanism proposed for explaining the correlation between meat intake and BC involves the generation of carcinogens, such as heterocyclic amines and polycyclic aromatic hydrocarbons by high-temperature cooking. This evidence claims the consumption limitation of meat. We reviewed the current literature on potential biological mechanisms underlying the impact of meat (red, white, and processed) intake on the increased risk of BC development and progression. Toward this purpose, we performed an online search on PubMed using the term “bladder cancer” in combination with “meat”, “red meat”, “white meat” or “processed meat”. Although some studies did not report any association between BC and meat intake, several reports highlighted a positive correlation between red or processed meat intake, especially salami, pastrami, corned beef and bacon, and BC risk. We speculate that a reduction or rather a weighting of the consumption of red and processed meat can reduce the risk of developing BC. Obviously, this remark claims future indications regarding food education (type of meat to be preferred, quantity of red meat to be eaten and how to cook it) to reduce the risk of developing BC. Further well-designed prospective studies are needed to corroborate these findings.

## 1. Introduction

Bladder cancer (BC) is the 10th most common malignant tumor diagnosed with a higher incidence in men worldwide [1,2]. Every year approximately 573,000 new cases are diagnosed, and more than 213,000 patients die of BC globally [3,4]. The BC incidence increases with age, and it is strongly associated with male gender, with men four times more frequently affected than women [5]. The main risk factors for BC are tobacco smoking and occupational exposure to aromatic amines (benzidine, 4-aminobiphenyl, 2-naphthylamine, 4-chloro-o-toluidine), polycyclic aromatic hydrocarbons and chlorinated hydrocarbons, accounting for 50% and 10% of all BC cases, respectively. The main sources of occupational exposure are industries involved in the production of dye, paint, metal and petroleum [6]. Radiation, pharmacologic agents (e.g., cyclophosphamide, pioglitazone) and arsenic exposure by drinking water also play an important role in BC etiology; in particular, the chlorination of drinking water is potentially carcinogenic. However, these factors do not fully explain BC occurrence [7]. Chronic Schistosoma infection is also responsible for BC onset and explains the high incidence of cancer in Egypt and other North African countries where this parasite is endemic [8].

Most chemical carcinogens need to be metabolically activated before becoming carcinogenic. Therefore, in addition to the intrinsic reactivity of its electrophilic derivatives, a chemical substance’s carcinogenic potential is also affected by the equilibrium between metabolic activation and inactivation reactions. Most known carcinogens are metabolized by cytochrome P-450 dependent monooxygenases.

Carcinogen exposure induces DNA damage that may result in DNA sequence alterations (mutations). Induced mutations could represent initiating events in cancer onset, when the damage occurs in oncogenes or tumor suppressor genes. Subsequently, once proliferation is induced by exposure to additional chemical agents, such as food factors, the DNA further undergoes mutations thus prompting cell transformation and tumor formation.

A relevant risk factor for BC development is polymorphism within genes coding for enzymes involved in xenobiotic biotransformation (i.e., Phase I and II reactions). In Phase I reactions, hydrophilic groups are introduced or exposed to render xenobiotics more hydrophilic and more easily excreted. In Phase II reactions (conjugation), xenobiotic is conjugated to a molecule (for example glucuronic acid), thus making it more water-soluble [9,10]. Some polymorphisms in genes encoding for Phase I (i.e., cytochrome p450) and Phase II components (i.e., glutathione S-transferase M1-GSTM1 and N-acetyltransferase 2-NAT2) have been associated with an increased BC risk [11]. However, no study has thoroughly evaluated the relationship between meat consumption and the genetic variations in the heterocyclic amines (HCA) metabolic pathways on BC risk [10].

Considering that nutrients or their catabolites are excreted through the urinary tract and come in contact with the bladder mucosa, diet could influence bladder carcinogenesis. Consequently, the identification of dietary factors associated with BC onset could aid in prevention [12,13].

Although literature data are controversial, a healthy diet could reduce the risk of many diseases including BC. In recent years, a protective effect of flavonoids and of the Mediterranean diet, in reducing the BC risk has been observed [14,15]. One possible explanation is that fruits and vegetables are rich in polyphenols, carotenoids and vitamins C and E, which work as antioxidants to prevent DNA oxidation by neutralizing reactive oxygen species [16].

Meat, conventionally classified as red, white or processed, despite being rich in saturated fat and cholesterol, provides the intake of a number of vitamins and minerals such as vitamin B, vitamin A, zinc and iron. In addition, it offers a broad array of proteins and essential amino acids [17,18]. In recent years, meat consumption has gradually increased worldwide, with the highest levels observed in Europe and the lowest in Southeast Asia and Africa. It has been estimated that in Europe, meat average intake for adults was 35 g/day, with the highest value in Austria (about 110 g/day) [19].

Throughout the past century, dietary practices have changed significantly. Europe has no exceptions, as nutritional preferences had significantly altered over time. Since the 1960s, meat consumption has constantly risen in the majority of nations, reaching a peak from the 1980s to the present, with the availability of meat products which increased of 204% between 1960 and 2010, compared with an increased meat consumption of up to 500% (1992–2016). If protein availability in the 1960s was predominantly plant-based today, up to 58% of protein availability comes from animal-derived foods; animal products currently make up the majority of protein sources (28 g of protein per person per day), contributing 30% of all calories consumed. Poultry and pig meats have seen the biggest increase in consumption among the various types of meat available on market [20].

The purpose of this manuscript is to evaluate the impact of red meat, especially processed meat consumption on the risk of BC development and progression.

## 2. Materials and Methods

### Literature Review

A comprehensive review of current literature on the association between meat consumption and BC risk was performed in the PubMed-Medline database using the term “bladder cancer” combined with one of the following terms: “meat”, “red meat”, “processed meat” and “white meat”.

The search was limited to articles analyzing human health, published in English. 

After the elimination of duplicates, the authors separately extracted data for further evaluation. Name of authors, journal and year, participant count per study, and accuracy of outcome prediction were taken from each study. All nutritional aspects, including dietary patterns, food groups, specific foods, macronutrients and micronutrients, were included. The results of the most recent publications were selected if the study provided adequate information about the number of patients, nutrients investigated, or risk estimated. The main characteristics of studies included in this narrative review are presented in Table 1 and Table 2.

## 3. Meat Consumption and Carcinogenesis: Exploring the Pathophysiology

In October 2015, the International Agency for Research on Cancer (IARC), analyzing epidemiological studies on the association between colorectal cancer and consumption of red or processed meat, classified the first as “probably carcinogenic to humans” (Group 2A) and the second as “carcinogenic to humans” (Group 1) [40].

Several mechanisms have been proposed to explain the carcinogenic potential of red and processed meat. Among them, the most accredited mechanisms involve the formation of chemical carcinogens during meat cooking (pan-fried, grilled/barbequed, oven-broiled, microwaved, other) and processing. Red meat and processed meat contain pro-carcinogenic compounds that are transformed into carcinogens, such as HCAs and polycyclic aromatic hydrocarbons (PAHs), during high-temperature or open-flame cooking [41]. PAHs are carcinogens because their metabolically activated intermediates form covalent bindings to DNA, leading to adduct formation [42]. Cytochrome P450 (CYPs) enzymes, CYP1A1 and CYP1B1 are involved in PAHs bioactivation [43]. More than 30 PAHs have been identified and, Benzo(a)pyrene (BaP) which is classified as a Group 1 human carcinogen, is the most toxic PAH in meat [17]. BaP, as well as all PAHs, acts as exogenous ligands of the nuclear translocator complex of the cytosolic aryl hydrocarbon receptor (AhR)–aromatic receptor nuclear translocator complex, increasing the expression of CYP450 family genes [43].

PAHs, in particular BaP, also exert a carcinogenic effect by downregulating or upregulating microRNA [44].

HCAs, similarly to PAHs, form covalent DNA adducts. Currently, about 25 HCAs, divided into amino-imidazo-azarenes and carbolines or pyrolytic HCAs, have been identified in cooked meat. HCAs formation depends on processing temperature; the optimal cooking temperature for HCA formation is between 150 and 200 °C [45]. The 2-amino-1-methyl-6-phenylimidazo(4,5b)pyridine (PhIP) and 2-amino-3,8-dimethylimidazo(4,5-f)-quinoxaline (MeIQx) are the HCAs most frequently found in red meat [46].

The final product of these reactions is the esterified N-hydroxy-HCA, obtained by CYP1A2 oxidation, subsequently acetylated and sulphated by acetyltransferases and sulfotransferases. The esterified N-hydroxy-HCA, through the nitrenium ion, can react with deoxy-guanosine of the DNA [47]. Additionally, a case-control study proposed that variations in the cytochrome P450 1A2-164 A/C (CYP1A2) or N-acetyltransferase 2 (NAT2) acetylator genotype may affect the relationships between consumption of red meat or meat-related mutagens and breast cancer risk [48].

Nitrate and nitrite, added to meat for their preservation and to enhance color and flavor, are precursors of N-Nitroso Compounds (NOCs), such as N-nitrosamines, alkylating agents that can react with DNA. Their production derives from the reaction between a nitrosating agent, produced by smoke, and a secondary amine, originating from lipid and protein degradation. Therefore, processed meat products can produce some NOCs during cooking, among which the most found are N-nitrosodimethylamine (NDMA), N-nitrosopiperidine (NPIP), N-nitrosodiethylamine (NDEA) and N-nitrosopyrrolidine (NPYR) [46].

In addition to exogenous NOCs derived from certain processed meats (e.g., grilled bacon), smoked fish, cheeses or beers, exposure to NOCs can be derived by endogenous mechanisms [49]. In fact, red meat contains heme iron, which increases the endogenous synthesis of NOCs and genotoxic free radicals in the colon, as highlighted by Bingham et al. [50,51].

Another possible explanation of the association between heme iron and cancer could be the damage caused by heme iron to the mucus barrier function thanks to the increase of mucin-degrading bacteria (e.g., *Akkermansia muciniphila*) [52]. Moreover, in addition to the direct damage on the gut microbiota, a high intake of red and processed meat can promote carcinogenesis through two compounds which can be useful to gut microbial metabolism: secondary bile acid and sulfur [53]. The first, produced by the anaerobic bacteria from bile acids, can increase oxidative/nitrosative stress and alter host metabolism; the second, metabolized by sulfur-reducing bacteria to hydrogen sulfide, can lead to direct DNA damage, epithelial hyperproliferation and inflammation [52].

### 3.1. The Role of Cooking and Meat Processing in Carcinogenesis

Cooking meat is a fundamental process to make it digestible, reduce contaminants (such as hormones, antibiotics, chemicals or metals) and give it flavor, juiciness and tenderness [54]. However, cooking meat can lead to the formation of chemicals harmful to human health by inducing chemical and physical changes [45]. 

Considering that meat cooking practices vary worldwide, currently available epidemiological studies are based on heterogeneous populations [55]. Consequently, it is difficult to evaluate the cooking impact on BC risk.

The amount and types of carcinogens present in cooked meat depend on cooking temperature, use of fat and direct or indirect contact with the heating source (Table 3).

The most widely used methods worldwide are barbecuing, grilling, deep-frying and pan-frying.

Steaming or stewing generates low levels of carcinogens, such as HCAs or PAHs, for the low temperatures used (about 100 °C), and the same applies to roasting, not so much for the temperature (up to 200 °C), but as there is limited direct contact with a warm surface. Instead, high-temperature cooking of meat, especially grilling, barbecuing or frying, and the exposition to hot surface or to direct flame causes amino acids and creatine reaction to form a variety of heterocyclic amines (HCAs) [53,54]. To reduce the formation of carcinogens, Felton et al. suggested some methods such as microwave pretreatment followed by the disposal of the resulting liquid before frying [55].

Processing meat can also reduce the risk of microbial contamination and ensure a more attractive appearance to products [56]. In fact, in addition to the substances produced by cooking meat, processed meat may contain additional toxic substances derived from various processing methods. Meat curing and smoking are the processes most involved in forming N-nitrosamines, formed by the reaction of a nitrosating agent and a secondary amine. In meat, nitrosating agents are gaseous nitrogen oxides, derived from smoking, and sodium nitrite derived from curing [57]. During smoking, wood pyrolysis can also lead to the generation of PAHs, however, in recent years, there have been changes to traditional processes to reduce the amount of these substances [58,59,60,61].

### 3.2. Red or Processed Meat and Bladder Cancer

Red meat refers to unprocessed mammalian muscle meat, such as beef, veal, pork, lamb, mutton, horse, or goat. It is a rich source of B vitamins (B6, B12, niacin, and thiamine), fatty acids, minerals such as iron and zinc, and proteins. The composition of meat varies depending on the animal’s species, sex, age, diet, climate, and activity during its growth; similarly, the livestock production system has a big impact on the meat’s nutritional value [62].

Several epidemiological studies examined the correlation between red or processed meat consumption and BC development. A case-control study demonstrated that the consumption of red meat at least 5 times a week induced a 2-fold increase OR than the consumption of meat less than once a week (OR = 1.8, 95% CI: 1.1–3.0) [25]. However, literature data are controversial. Although in case-control study, Crippa et al. observed that red meat consumption was associated with BC risk, no association was observed in prospective studies (RR = 1.51, 95% CI: 1.13–2.02). In addition, Fei Li et al. found no significant overall association between red meat intake and BC incidence, but a 25% higher risk of BC for red meat in the populations of the American continent [21]. 

In both studies, it was evident that the BC risk was positively correlated with the consumption of processed meat (salting, fermentation, smoking or other processes). In particular, a 20% increase in the BC risk is associated with an increase of 50 g of processed meat per day (RR = 1.20, 95% CI: 1.06–1.37) [26]. The prospective study performed by Xu et al. found a positive association between processed red meat intake and BC risk, after adjusting for confounders (HR = 1.47, 95% CI: 1.12–1.93) [38].

Another case–control study by Balbi et al. showed that salted meat intake is associated with a greater risk of BC development with an OR of 2 that further doubles if the quantities of taken meat increases or subjects are long-term smokers (OR =18.3, 95% CI: 4.6–71.9) [22]. These results were confirmed by another case–control study conducted by De Stefani et al. (OR = 2.23, 95% CI: 1.63–3.04) [27].

Rosato et al. also found a moderate risk of hormone-dependent tumors in women with an elevated processed meat intake, suggesting that a link between meat and women cancers could be related to sex steroid hormone levels. In particular, a significant 20% excess risk of BC for 10 g intake daily increase in women was observed, while was not possible to state the role of processed meat consumption in the etiology of BC in men (OR = 1, 95% CI: 0.94–1.06) [28]. Furthermore, a case-control study conducted in Japan, revealed a positive association between BC risk and bacon, ham, and sausage intake only in men while no associations were observed in women [63]. Before these findings, two studies showed no difference between men and women [24,30].

Catsburg et al. found that a diet rich in salami/ pastrami/corned beef or liver may increase the BC risk, and observed that high nitrate intake and consumption of large amounts of processed meats have a synergistic effect (OR = 1.76, 95% CI: 1.09–2.85) [23].

An interesting prospective study, conducted by Dianatinasab et al. containing data from 11 cohort studies showed an increased risk of BC for high organ meat (liver and other offal) intake, (HR comparing highest with the lowest tertile: 1.18, 95% CI: 1.03, 1.36, p-trend = 0.03). While a null association between red and processed meat consumption was observed. [37]

Although the overall association was not statistically significant, in 2 large prospective studies Michaud et al. showed an elevated BC risk among men and women who consumed 5 servings of bacon/week compared with those who consumed none (RR = 1.59, 95% CI: 1.06–2.37) [24].

About the cooking methods, an Italian multicenter case-control conducted by Di Maso et al. has found that the consumption of meat increased the risk of BC, especially when it is roasted or stewed.

Despite the above studies showed an association between processed or red meat and BC, for many authors the risk of developing BC does not change with the increase in consumption of processed or red meat [31,32].

Furthermore, three other studies found no association between meat cooking techniques and BC risk [29,33,34,35,36,64].

### 3.3. White Meat and Bladder Cancer

The term “white meat” is typically used to identify light-colored meat before and after cooking such as poultry (e.g., chicken, turkey), and rabbit. Currently, few studies analyzed the associations between white meat consumption and BC, so it is difficult to evaluate its role in BC development.

Nevertheless, an increased white meat intake has been negatively associated with some cancer types [65]. This is consistent with the findings of Dianatinasab ‘s study, which showed a non-significant association between poultry and BC risk (RR = 0.77; 95% CI: 0.48–1.06) [37]. However, the NIH-AARP Diet and Health study reported a statistically significant decrease in BC risk associated with 10 g per day of white meat consumption (HR = 0.86; 95% CI: 0.76–0.98) [39]. Among the possible explanations, white meat, compared to red meat, releases less mutagenic substitutes during the cooking and contains less saturated fats and heme iron, potential inducers of oxidative stress and DNA damage [66]. In addition, white meat is a source rich of polyunsaturated fatty acids (PUFAs) that seem to prevent carcinogenesis through their anti-inflammatory moieties. Among PUFAs, Omega-3 (n-3) in fact inhibits the synthesis of pro-inflammatory cytokines, such as IL-1 and TNF [67]. However, results of epidemiological studies on the relationship between PUFAs intake and BC risk are misleading: some studies showed a null association, while others produced an inverse association [68,69].

Conversely, Michaud et al. affirmed that an increased intake of chicken without skin was positively associated with BC (RR = 1.52; 95% CI: 1.09–2.11), compared to chicken with skin (RR = 1.10; 95% CI: 0.86–1.41) [24]. These results could be explained by high heterocyclic amine concentrations in chicken cooked without skin, compared to chicken with skin, under the same cooking conditions [70].

## 4. Future Directions

Is it possible to eliminate meat from the diet? To answer this question, it would be relevant to better understand eating patterns that include varying amounts of red meat to promote overall health. Current WCRF-AICR recommendations suggest eating more than about three portions of red meat per week, equivalent to about 350–500 g. This amount ensures a balance between the advantages (source of essential micro- and macronutrients) and the disadvantages (an increased cancer risk) [71].

Some methods, such as microwave pretreatment or the use of organosulfur compounds contained in garlic or onion, were advocated as potential measures aimed to decrease the HAAs levels in cooked meats [72].

Surely, the eating habits of the various cultures have strongly conditioned the available data. Nevertheless, for clinical practice purposes, a more varied diet containing a mixture of pulses and cereals is certainly encouraged for obtaining adequate protein requirements [71].

Epidemiological studies should be increased and focused to better examining the association between red meat intake and several diseases, such as BC. In this way, consistent and rigorous results could be achieved to define recommended meat consumption (i.e., the frequency expressed in day, week or month) on the basis of national intake data and consequently inform the dietary habits of the population [73]. The role of the cooking method should also be deepened, and the misleading variables that can induce wrong associations should be identified.

It would be relevant to evaluate the meat impact in maintaining lean mass and appropriate body weight throughout the life, thus helping to reverse the global trend towards overweight and obesity which has increased especially among children. Finally, it is relevant to make additional efforts to completely comprehend how red meat influences food security, illness burden, and nutritional status in developing countries.

## 5. Conclusions

This review identified meat as a possible risk factor for BC. Associations between meat intake and BC differed according to cooking method, temperature applied, and type of meat consumed; in some studies, white meat consumption was negatively related to BC.

These data are certainly interesting and confirm the hypothesis that Western dietary patterns contribute to BC etiology and prognosis. Further studies are needed to determine the mechanisms responsible for the Western dietary pattern’s effect on BC carcinogenesis and to discover the Western dietary pattern components predominantly associated with BC risk. In addition, it should be clarified the diet type recommended for reducing BC incidence. The progress in our knowledge on the link among meat intake and BC risk could be useful for education campaigns aimed at encouraging dietary habit changes providing valuable benefits for public health.

## Figures and Tables

**Table 1:** Characteristics of studies that analyzed red meat or processed meat consumption and bladder cancer.

Type of Study	Number of Patients Enrolled	Exposure Variable (Definition)	OR	RR	HR	95% CI	Years of Follow-Up (Median)	Adjustments	Remarks	References
MA	1,520,308	Total red meat	-	1.15	-	0.97–1.36	-	Age, sex, smoking, geographic region, total energy, BMI, study design	increased by 25% the risk of BC for red meat in the population of USA	[21]
CC	744	Salted meat	18.3	-	-	4.6–71.9	1	Age, sex, smoking, BMI, total calories, education, urban/rural status and ‘mate’ drinking	the risk was especially elevated among long-term smokers	[22]
CC	1660	Salami/pastrami/corned beef or liver	1.76	-	-	1.09–2.85	9	Age, gender, race/ethnicity	associated with risk of BC, particularly among nonsmokers	[23]
CO H	135,893	Beef, pork or lamb	-	1.59	-	1.06–2.37	22	Age, smoking, caloric intake, geographic region and total fluid intake	elevated risk among men and women who consumed 5 servings of bacon/wk	[24]
CC	956	Red meat	1.8	-	-	1.1–3.0	3	sex, age, smoking status, smoking duration, smoking amount	consumption of red meat at least 5 times a week induced a 2-fold increase OR than the consumption of meat less than once a week	[25]
MA		processed meat (salting, fermentation	-		-		-	Age, gender, smoking		[26]
	1,066,027	Smoking or other processes		1.20		1.06–1.37			A 20% increase in the risk of BC is associated with an increase of 50 g of processed meat per day	
CC	13,050	Salted meat	2.23	-		1.63–3.04	7	Age, sex, residence, education, family history of BC, high-risk occupation, body mass index, years smoked, and total energy intake	intake of salted meat is associated with a greater risk of BC	[27]
CC	3149	processed meat	1.23	-	-	1.03–1.47	7	sex, age, education, smoking, alcohol drinking, BMI, vegetable and fruit consumption, and total energy intake	a moderate risk of hormone-dependent tumors in women with an elevated processed meat intake	[28]
CC	1355	processed meat (stewed and roasted)	1.57 (read meat) 1.47 (stewed) 1.41 (roasted)	-	-	1.07–2.31 1.03–2.09 1.00–1.99	11	sex and smoking	consumption of meat increased the risk of BC, especially when it is roasted or stewed	[29]
CO H	82,002	Red meat (beef, pork, meatballs, hamburger, veal and kidney or liver)	-	-	1.05 (Total meat) 1.0 (Red meat) 1.01 (Processed meat)	0.71–1.55 0.71–1.41 0.80–1.28	9	Age, sex, smoking, education and total energy intake	no association between the intake of total or any specific type of meat	[30]
CO H	481,419	Meat intake	-	-	1.06	0.99–1.13		Total energy intake, smoking, education and BMI	no overall association between intake of red meat and BC	[31]
MA	7022	Meat intake	-	1.08	-	0.82–1.42	-	Smoking	a diet with a high content in fruits and vegetables may prevent bladder cancer	[32]
MA	-	Meat intake	-	1.04	-	0.80–1.27	-	Age, sex and smoking	A low increase in the risk of BC with high processed meat consumption	[33]
CO H	1,922,817	Meat intake	-	-	1.22	0.96–1.54	9	Age, sex, smoking, vegetables beverages fruit and total energy	possible increased risk of BC with PhIP (2-amino-1-methyl-6-phenylimidazo [4, 5-*b*]pyridine) exposure	[34]
CC	2589	Meat and processed meat intake	1.28 (Meat) 1.41 (Processed meat)	-	-	1.00–1.65 1.08–1.84	3	Gender, age, education, race, smoking, BMI, and total energy	meat cooking methods are not associated with BC risk	[35]
CC	273	Meat intake	-	1.20	-	0.7–2.1	-	Age, sex, smoking, energy	an increased cancer risk when the intake of heterocyclic amines is high (above 1900 ng daily)	[36]
COH	518,545	Meat intake	-	-	1.18	1.03–1.36	-	age, sex, smoking, total energy intake, and vegetables and fruits consumption	meat consumption may be associated with BC development	[37]
COH	101,721	Processed meat	-	1.47	-	1.12–1.93	12.5	age, sex, race, BMI, smoking, alcohol drinking, total energy intake, and family history of any cancer type	a higher risk of BC is associated with intake of processed red meat	[38]

CC: Case-Control; COH: Cohort study; MA: Meta-analysis; BC: bladder cancer.

**Table 2:** Characteristics of studies that analyzed white meat consumption and bladder cancer.

Type of Study	Number of Patients Enrolled	Exposure Variable (Definition)	OR	RR	HR	95% CI	Years of Follow-Up (Median)	Adjustments	Remarks	References
COH	2296	White meat (poultry and fish)	-	-	0.83 (Poultry) 1.13 (Fish)	0.73–0.96 0.99–1.29	9.1	Age, sex, education, BMI, race, smoking, total energy, alcohol drinking	a decrease in BC risk associated with 10 g/per 1000 kcal in white meat consumption	[39]
COH	518,545	Meat intake	-	0.77	-	0.48–1.06	-	age, sex, smoking, total energy intake, and vegetables and fruits consumption	No association between poultry and BC	[37]
COH	135,839	Chicken (without skink)	-	1.52	-	1.09–2.11	22	Age, smoking, caloric, geographic region and total fluid intake	a positive association was detected for intake of chicken without skin, but not for chicken with skin	[24]

**Table 3:** The different cooking methods and the influence on the production of potential carcinogens.

Cooking Method	Definition	Temperature	Effect
Stewed	cooked by boiling or simmering in the liquid contained in an enclosed vessel	Around 100 °C	Generate much lower levels of HCAs or PAHs
Boiled	cooked in boiling liquid		
Steamed	cooked by steam, in pressure cooker or cooked suspended above boiling water		
Barbecued	cooked on grill bars over burning charcoal, wood or gas	200 °C or more	The exposition to a hot surface or to direct flame causes amino acids and creatine to react to form a variety of HCAs
Grilled	cooked rapidly without moisture, on grill bars under or over intense direct heat		
Fried	cooked in heated fat, usually over a direct source of heat		

HCAs: heterocyclic amines; PAHs: polycyclic aromatic hydrocarbons. Table compiled using data from the EPIC study [56].

## Abbreviations

BC: bladder cancer; HCA: heterocyclic amine; PAH: polycyclic aromatic hydrocarbon; BaP: Benzo(a)pyrene; NOC: N-Nitroso Compound; RR: relative risk; OR: odds ratio; PUFAs: polyunsaturated fatty acids.
